# MiR-376a-3p increases cell apoptosis in acute myeloid leukemia by targeting MT1X

**DOI:** 10.1080/15384047.2022.2054243

**Published:** 2022-03-22

**Authors:** Xiangke Xin, Zheng Xu, Jia Wei, Yicheng Zhang

**Affiliations:** a Department of Hematology, Tongji Hospital, Tongji Medical College, Huazhong University of Science and Technology, Wuhan, Hubei, China; b Department of Hematology, Xinyang Hospital Affiliated to Zhengzhou University, Xinyang, Henan, China

**Keywords:** AML, metallothionein 1X, miR-376a-3p, proliferation, apoptosis

## Abstract

Metallothioneins (MTs) are a group of low-molecular weight cysteine-rich proteins that play vital roles in oxidative stress, metal homeostasis, carcinogenesis and drug resistance. However, few studies have analyzed the roles of MTs in acute myeloid leukemia (AML). In this study, we revealed that the expression of metallothionein1X (MT1X), a main isoform of MTs, was highly expressed and acted as a candidate of prognostic indicator in AML patients. In vitro cell function experiments verified that silencing MT1X inhibited the proliferation of AML cells, sensitized cells to doxorubicin, and increased their apoptosis. We also showed that the downregulation of MT1X expression suppressed nuclear factor-κB (NF-κB) signaling by reducing p65, p-IκB-α, and downstream effectors. Elevated p65 and MT1X levels were indicators in AML. Moreover, we revealed that miR-376a-3p had binding sites with 3ʹ-UTR of MT1X, suggesting that MT1X was negatively regulated by miR-376a-3p. Cell functional assay results indicated that miR-376a-3p overexpression significantly inhibited the proliferation, arrested the AML cells in the G0/G1 phase and induced cell apoptosis. The rescue experiments further confirmed that miR-376a-3p could reverse the promotion of MT1X overexpression on the progress of AML cells. Taken together, our results revealed that elevated MT1X expression might be involved in the mechanism underlying AML progression, indicating that the miR-376a/MT1X axis might serve as a promising novel target for the effective treatment of patients with AML.

## Introduction

Acute myeloid leukemia (AML), the most common type of acute leukemia in adults, is a heterogeneous myeloid malignancy.^1^ It is characterized by an accumulation of immature blast cells due to deficient maturation, uncontrolled proliferation, dysregulated apoptosis and the inhibition of normal hematopoiesis.^2^ In recent years, with the development of chemotherapy, hematopoietic stem cell transplantation, cell therapy, biological immunotherapy, and gene targeted therapy, the complete response rate and recurrence-free survival rate of AML patients have improved than before, but there are still a majority of patients with drug resistance and relapse after remission. Consequently, it is indispensable to discover and develop new strategies for the accurate diagnosis and effective treatment of AML.

Metallothioneins (MTs) are a family of low-molecular weight (ranging from 6 to 7 kDa), cysteine-rich cytosolic proteins that play a vital role in metal ion homeostasis and detoxification, metal donation to different enzymes, angiogenesis, apoptosis, cell differentiation, and drug resistance as well as carcinogenesis.^3–7^ For example, MT1F transfection into colon cancer cells could decrease cell proliferation, colony formation, migration, invasion, and adhesion and increase cell apoptosis.^8^ MT2A gene expression could enhance the chemosensitivity to doxorubicin.^9^ The overexpression of MT1G sensitized colorectal cancer cells to the chemotherapeutic agents oxaliplatin and 5-fluorouracil, which might have been mediated by the activation of p53 and repression of NF-κB activity.^10^ MT1X, as a tumor suppressor, was involved in the progression and metastatic capacity of hepatocellular carcinoma.^11^ Microarray-assisted pathway analysis identified MT1X & NF-κB as mediators of TCRP1-associated resistance to cisplatin in oral squamous cell carcinoma.^12^ These studies have suggested that MTs participate in the process of carcinogenesis and drug resistance in solid tumors. However, relatively little is known about the role of MTs in AML.

Duval et al. showed that overexpression of MT1 prevented apoptosis of embryonic stem cell-derived differentiated cells.^13^ Maghdooni Bagheri et al. revealed that the more mature hematopoietic precursor cell lines K562, DAMI, and MEG01 cells had higher MT levels than the immature ELF153 cells.^14^ Overexpression of MT2A in megakaryocytic DAMI cells caused increases in the cell size, intracellular granulation, and levels of megakaryocytic-specific CD41 and CD42 with arrest of cell proliferation.^15^ MT was expressed in leukemic cells from 68% of cases with newly diagnosed AML. The levels of MT expression in the relapse patients were higher than those in the remission patients.^16^ The expressions of the MT1A and MT1G genes were significantly inversely correlated with the hematopoietic transcription factor PU.1 gene in 43 primary acute AML specimens.^17^ These studies indicated that increased MTs expression represented a poor prognostic marker for AML. Nevertheless, the molecular mechanism of MTs in AML remains abstruse.

In our research, the aim was to detect the key functions of MTs in the proliferation apoptosis and to determine the biological roles and regulatory mechanism in AML cells, thereby offering a better understanding of the MT’s function in AML to develop it as a promising diagnostic and therapeutic target for this disease.

## Results

### MT1X is highly expressed in AML

**Figure 1.**
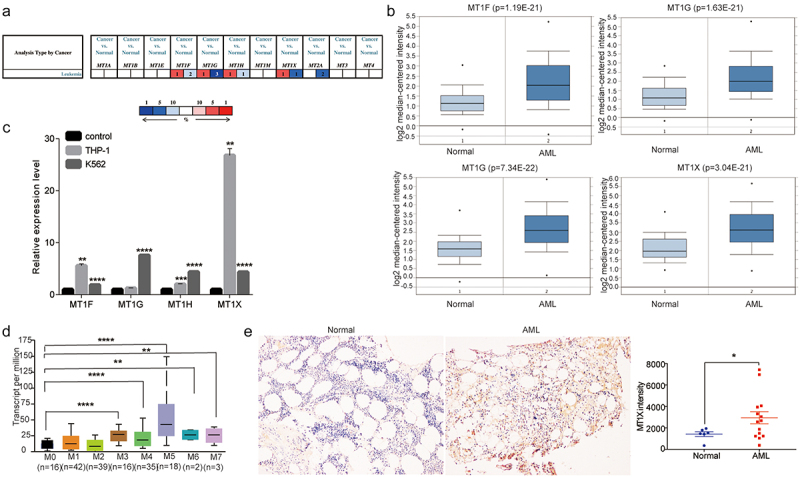
MT1X is highly expressed in AML. (a) The figure showed the numbers of datasets with statistically significant mRNA over-expression (red) or downregulated expression (blue) of MT (ONCOMINE); (b) The expression of MT1F, MT1G, MT1H, MT1X were low in normal samples and high in AML samples (ONCOMINE); (c) mRNA expressions of MT1F, MT1G, MT1H, MT1X in normal samples and AML cell lines (THP1 and K562); (d) The expression of MT1X differed in several AML subtypes classified as FAB (UALCAN); (e) Immune histochemical staining (brown) of MT1X in bone marrow samples from 6 healthy controls and 14 AML patients. Blue stain represented hematoxylin nuclear staining.

Note: *Indicates *P < .05; **P < .01; ***P < .001; ****P < .0001.

Eleven isoforms of MTs were retrieved using the ONCOMINE database. We first explored the transcriptional levels of MTs in leukemia and normal peripheral blood mononuclear cells with ONCOMINE (Figure 1(a)). Based on the data from ONCOMINE, the transcriptional levels of MT1F, MT1G, MT1H and MT1X in AML were considerably highly expressed in AML than normal samples (Figure 1(b)). Then, we found that the mRNA levels of MT1F, MT1G, MT1H, and MT1X were significantly elevated in THP1 and K562 cells vs. normal samples. Moreover, MT1X in THP1 had the highest expression among the four isoforms, where the mRNA level was significantly elevated with a fold change of 26.81 (Figure 1(c)). We also assessed the expression levels of MT1X in several AML subtypes classified as FAB through UALCAN. The expression of MT1X was elevated in all subtypes of AML and was the highest in M5 (Figure 1(d)). Finally, we compared MT1X expression in normal bone marrow tissues and AML samples. MT1X proteins localized predominantly to the cytoplasm and stroma of the AML cells and were sparse in the nucleus. Higher levels of MT1X were found in AML compared with normal bone marrow tissues (Figure 1(e)). In conclusion, we deemed that MT1X was highly expressed in AML and might serve as an oncogene in AML.

### Knockdown of MT1X inhibits the proliferation, increases apoptosis, and sensitizes cells to doxorubicin

**Figure 2.**
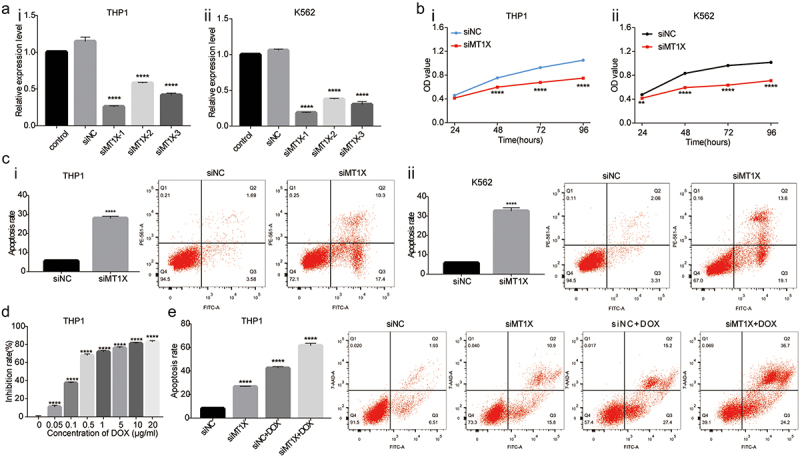
Downregulation of MT1X inhibits the proliferation, increases apoptosis, and sensitizes cells to doxorubicin. (a) mRNA levels of MT1X in MT1X-silenced THP1 and K562 cells; (b) Proliferation of THP1 and K562 cells were analyzed by using CCK8 assays at 24, 48, 72 and 96 h after transfection with siRNA MT1X or control siRNA (siNc); (c) Apoptosis analysis was determined by Annexin-PI assay in MT1X-silenced THP1 and K562 cells; (d) CCK8 assay was used to analyze the proliferation inhibition rate of THP1 cells treated with doxorubicin (DOX) at different concentrations (0, 0.05, 0.1, 0.5, 1, 5, 10, 20 μg/ml) for 48h;(e) Apoptosis was determined by flow cytometry via Annexin-PI staining in THP1 cells transfected with siRNA MT1X or treated with DOX or a combination of both.

Note: *Indicates *P < .05; **P < .01; ***P < .001; ****P < .0001.

The mRNA expression of MT1X was distinctly reduced in THP1 and K562 cells transfected with three sets of siMT1X (Figure 2(a)). siMT1X-1, the most significant decrease, was selected for follow-up experiments. The results of CCK8 (Figure 2(b)) and Annexin-PI (Figure 2(c)) assays revealed that decreased MT1X expression resulted in the inhibition of the proliferation activity and the promotion of the apoptosis rate. Different concentrations of doxorubicin (DOX) were added into the THP1 cells and resulted in a dose-dependent inhibition of the proliferation in the THP1 cells (Figure 2(d)). The IC50 of DOX on inhibiting THP1 proliferation was 0.372 µg/ml. To examine the role of MT1X in drug resistance, the THP1 cells were transfected with siRNA MT1X or treated with DOX or a combination of both. The results exhibited that the apoptosis rate of THP1 cells transfected with siRNA MT1X and treated with DOX was significantly higher, indicating that downregulation of MT1X sensitized cells to doxorubicin (Figure 2(e)).

### Downregulation of MT1X represses NF-κB activity

**Figure 3.**
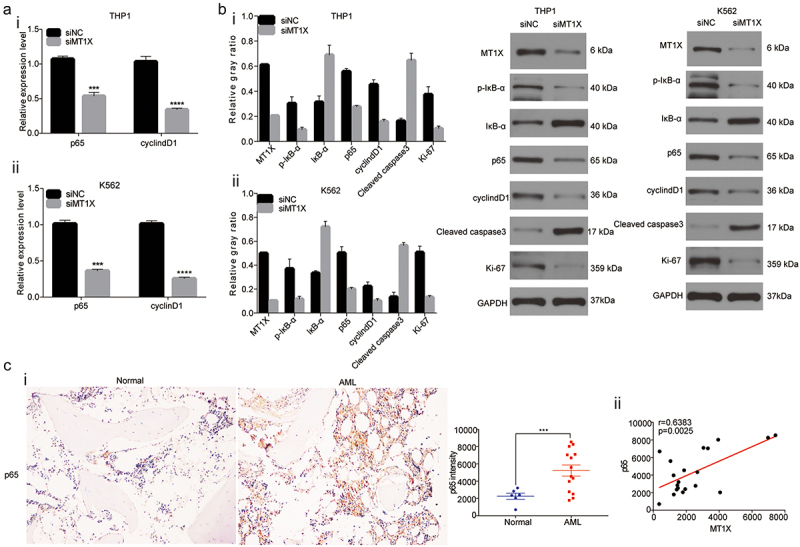
NF-κB pathway is suppressed in MT1X-silenced THP1 and K562 cells. (a) qRT-PCR was applied to determine the expression of p65 and cyclinD1 in THP1 and K562 cells transfected with siRNA MT1X or control siRNA. (b) Western Blot was applied to determine the expression of the key molecules in NF-κB signaling, such as p65, p-IκB-α, IκB-α, and the downstream effector of NF-κB signaling-cyclinD1. (c) Immune histochemical staining (brown) of p65 in bone marrow samples from 6 healthy controls and 14 AML patients. Blue stain represented hematoxylin nuclear staining. Measurement of staining intensity comparing bone marrow samples versued AML samples.

Note: *Indicates *P < .05; **P < .01; ***P < .001; ****P < .0001.

MTs have been demonstrated to interact with NF-κB and mediate its antiapoptotic effects.^18,19^ However, the role of MT1X as an ally or enemy in NF-κB signal pathway is still in dispute. We studied whether the NF-κB pathway participated in the enhanced role of MT1X in AML. MT1X and the main genes in NF-κB signaling, such as p65, IκB-α, p-IκB-α, and the downstream effector of NF-κB signaling-cyclinD1 were detected in our study. As shown in Figure 3(a), knockdown of endogenous MT1X in THP1 and K562 cells led to downregulation of p65 and cyclinD1 transcription expression. The relative protein levels of p65 and cyclinD1 were consistent with mRNA transcripts. The protein levels of p-IκB-α and Ki-67 were decreased, while the IκB-α and cleave caspase3 protein were increased in those THP1 and K562 cells transfected with siRNA MT1X (Figure 3(b)). Downregulation of MT1X increased IκB-α, which suppressed the NF-κB pathway, resulting in the typical apoptosis of cells resulting from suppression of NF-κB activation, accompanied by G0/G1 arrest. Therefore, we speculated that downregulation of MT1X induced cell apoptosis via repressing NF-κB signaling cascade in AML. Next, we studied levels of p65 in bone marrow samples from 6 healthy controls and 14 AML patients. P65 proteins localized largely to the nucleus and cytoplasm of the AML cells and were sparse in the stroma (Figure 3(c)). The expression of p65 increased in AML samples than healthy controls, and the expression of MT1X and p65 exhibited a strong positive correlation (Spearman correlation analysis, r = .0.6383 p = .0025) (Figure 3(c)).

### MT1X is a target gene of miR-376a-3p and negatively regulated by miR-376a-3p

**Figure 4.**
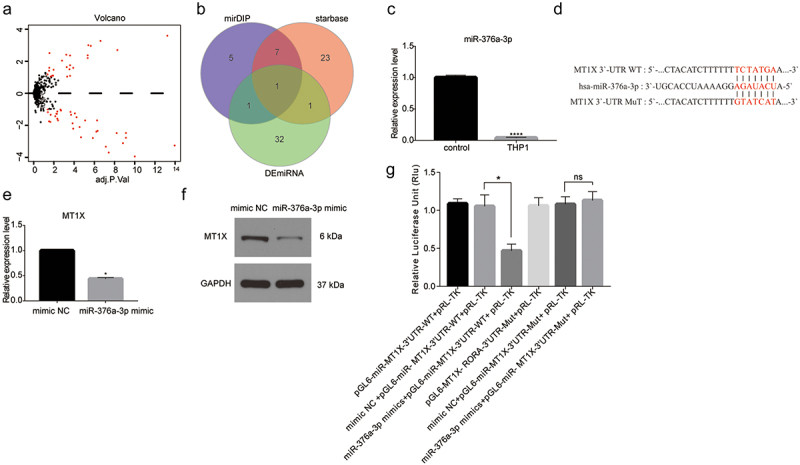
miR-376a-3p targeted downregulates MT1X. (a) DEmiRNA volcano map of normal group and AML group in GSE142699 datasets (GEO); (b) Venn diagram was applied to predicate upstream downregulated DEmiRNAs for MT1X; (c) The miR-376a-3p expression was highly expressed in normal sample and lowly expressed in THP1 cell line; (d) Binding sites of miR-376a-3p and 3 ‘UTR of MT1X; (e and f) qRT-PCR and western blot were applied to determine the effects of miR-376a-3p expression on MT1X mRNA and protein levels; (g) The dual-luciferase reporter gene was performed to detect the targeted binding of miR-376a-3p and MT1X.

Note: *Indicates *P < .05; **P < .01; ***P < .001; ****P < .0001.

To explore the molecular mechanism of MT1X in AML, we performed bioinformatics analysis of its upstream regulatory factors. First, based on the data in GSE142699 data sets, a total of 58 DEmiRNAs were acquired by “edgeR” for differential analysis (Figure 4(a)). Then, we utilized the target prediction website, mirDIP and starBase, to predicate the upstream miRNAs of MT1X, and the potential regulatory factor miR-376a-3p of MT1X was acquired through the intersection of the downregulated 35 DEmiRNAs (Figure 4(b)). The result of qRT-PCR displayed that miR-376a-3p was significantly low-expressed in the THP1 cell line (Figure 4(c)). Bioinformatics analysis suggested that miR-376a-3p and MT1X had targeted binding regions (Figure 4(d)). To confirm the targeted regulatory relationship between miR-376a-3p and MT1X, the miR-376a-3p mimic group or NC group was transfected into the THP1 cells, the results of qRT-PCR and western blot indicated that the expression of MT1X was significantly downregulated when miR-376a-3p was overexpressed (Figure 4(e,f)). Furthermore, the Dual-luciferase reporter gene assay was performed, and the result displayed that miR-376a-3p mimic significantly reduced luciferase activity in the MT1X-WT group but had no effect on that of the MT1X-Mut (Figure 4(g)). Therefore, the results revealed that MT1X was a direct target gene of miR-376a-3p and was negatively regulated by miR-376a-3p in AML.

### Overexpression of miR-376a-3p inhibits proliferation, arrests in the G0/G1 phase and induces apoptosis of AML cells which can be reversed by MT1X

**Figure 5.**
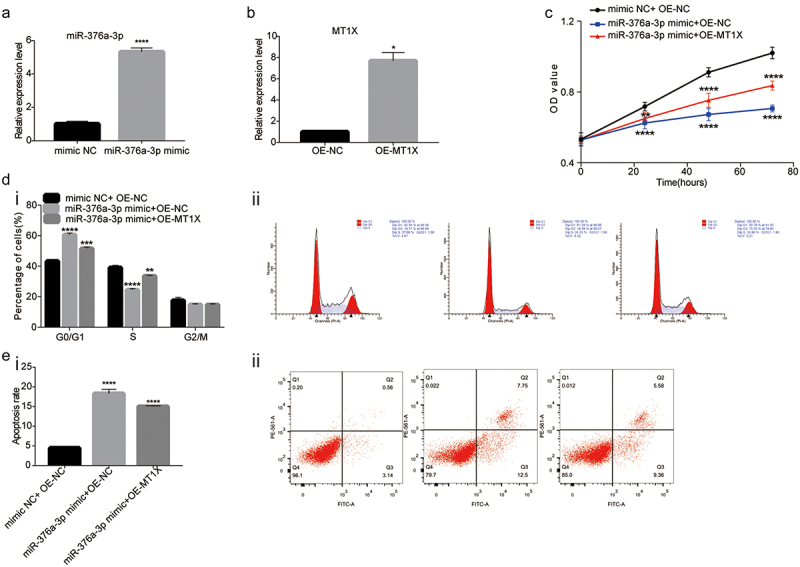
Upregulation of miR-376a-3p inhibits AML development, which can be reversed by MT1X. (a and b) Expression of miR-376a-3p and MT1X were detected by qRT-PCR in THP1 cells transfected with miR-376a-3p mimic or OE-MT1X; (c) CCK8 assay was applied to detect the cell viability at 0, 24, 48 and 72 h in THP1 cells transfected with miR-376a-3p mimic or OE-MT1X or a combination of both; (d and e) The cell cycle and the apoptosis of THP1 cells were analyzed by flow cytometry.

Note: *Indicates *P < .05; **P < .01; ***P < .001; ****P < .0001.

Due to the regulation of miR-376a-3p on MT1X, we further studied whether miR-376a could affect the cell functions in AML. First, the miR-376a-3p mimic group or oe-MT1X was transfected into the THP1 cells to examine the overexpression efficiency of miR-376a-3p and MT1X (Figure 5(a)). The results of CCK8 (Figure 5(b)) and flow cytometry (Figure 5(d)) assays displayed that compared with the NC group, the upregulation of miR-376a-3p significantly inhibited proliferation and induces apoptosis of THP1 cells, while overexpressed MT1X would attenuate the inhibitory effects of miR-376a-3p overexpression in AML cells. Furthermore, the result of cell cycle indicated that upregulation of miR-376a-3p blocked cells in the G0/G1 phase to inhibit cancer cell proliferation (Figure 5(c)). Taken together, our study indicated that miR-376a-3p inhibited cell function by inhibiting MT1X expression in AML.

## Discussion

In this research, we verified that MT1X was distinctly upregulated in AML. Cytological studies displayed that silencing MT1X regressed the proliferation, increased apoptosis and sensitized cells to doxorubicin in AML cells. We also showed that knockdown of MT1X suppressed NF-κB signaling by reducing p65, p-IκB-α and downstream effectors. Elevated p65 and MT1X levels were indicators in AML. Mechanism studies indicated that miR-376a-3p could regulate the expression of MT1X.

MTs have been observed to participate in a series of cellular processes, such as redox and metal homeostasis, immunomodulation, anti-inflammatory reactions, and tumor development and progression.^4,5,7,20–22^ In this research, through the analysis of ONCOMINE database, we found distinct differences in MT1F, MT1G, MT1H, MT1X expression in normal group and AML group. MT1X had the highest expression among the four isoforms in AML cell line vs normal sample, where the mRNA level was significantly elevated with a fold change of 26.81. Studies have displayed that MT1X may serve as a candidate of prognostic indicator and inhibits the progression and metastasis of hepatocellular carcinoma.^11^ Therefore, we utilized the UALCAN portal to perform the expression of MT1X in AML clinical subtypes classified as FAB in TCGA-LAML dataset, and found that the expression of MT1X was elevated in all subtypes of AML and was the highest in M5. Moreover, we also performed knockdown of MT1X expression in THP1 and K562 cells to confirm their effects on cell function and discovered that silencing MT1X significantly inhibited the proliferation, increased apoptosis and sensitized cells to doxorubicin. In conclusion, our results further verified that MT1X was associated with AML progression and an unfavorable prognosis in AML.

NF-κB has a key role as a pivotal link between inflammation and cancer.^23^ Recently, NF-κB inhibitors have been severed as a new target for tumor therapy.^24,25^ Numerous studies displayed that MTs had been shown to interact with NF-κB and mediated its antiapoptotic effects.^18,19^ In this study, we demonstrated that knockdown of endogenous MT1X led to downregulation of p-IκB-α and cyclinD1 but induced IκB-α upregulation, which was in line with the typical apoptosis of AML cells resulting from regression of NF-κB activation. The protein level of Ki-67 was decreased as well as the cleaved caspase 3 protein was increased in those THP1 cells transfected with siRNA MT1X, which demonstrated that MT1X was related to the growth of AML cells. Next, we observed that MT1X and p65 protein strongly expressed in AML samples but less in normal samples. Their expression exhibited a strong positive correlation. Taken together, elevated p65 and MT1X levels were indicators in AML.

MicroRNA is a small non-coding RNA molecule playing a crucial role in the biological process of cell cycle regulation, proliferation, apoptosis, and differentiation.^26^ It is predominantly through directly binding to the 3´UTR of the target gene mRNA and inducing translational inhibition or post-transcriptional degradation.^27,28^ For the purpose of further exploring the upstream regulatory mechanism of MT1X, we utilized bioinformatics to analyze miRNAs in GSE142699 to obtain miR-376a-3p, which had targeted binding sites with MT1X and was obviously downregulated in AML. The results of some experiments showed that miR-376a-3p could target the downregulation of MT1X in the AML cell line, predicting that it might be as a tumor depressor regulating the process of AML.

miR-376a-3p has been involved in several types of cancers, suggesting pivotal roles in various cell processes, including cell proliferation, apoptosis, invasion and metastasis.^29–32^ miR-376a-3p, induced by TTN-AS1, mitigates the effects on colorectal cancer cell proliferation and invasion, which is a target of TTN-AS1,^33^ miR-376a-3p mitigates the development of glioma by negatively regulating KLF15.^34^ In addition, miR-376a-3p inhibits cell proliferation and invasion of osteosarcoma cells by directly targeting SATB1^35^ In the present study, we confirmed that miR-376a-3p could affect the function of AML cells by regulating MT1X at the cellular level. Our results revealed that overexpression of miR-376a-3p evidently inhibited the proliferation, arrested in the G0/G1 phase and induced apoptosis of AML cells, and its suppressive effect could be reversed when MT1X was overexpressed, which were consistent with the trend of miR-376a-3p effects in other tumors. In the future, we can also utilize the miR-376a-3p/MT1X axis as a molecular target for the treatment of AML, thus improving the treatment and prognosis of AML patients.

As the key protein of anti-apoptosis, MT1X plays a vital roles in the proliferation of cancer cells. Our research investigated the mechanism of upregulated MT1X in regulating the malignant process of AML as a target gene of miR-376a-3p. In the immediate future, not only can MT1X act as an AML diagnostic marker, but also they can improve the therapeutic effect of AML patients. Taken together, this research provides a new theoretical basis for molecular targeted therapy of AML in the future.

## Material and methods

### Bioinformatics

The GSE142699 (Normal: 24, Tumor: 24) data set was downloaded from the Gene Expression Omnibus (GEO) database (https://www.ncbi.nlm.nih.gov/geo/). The “sva” package was used to calibrate the data set in batches. To acquire the differentially expressed miRNAs (DEmiRNAs), the differential analysis between the normal group and the AML group was performed using “edgeR” package (|logFC|>1, padj <0.05). mirDIP (http://ophid.utoronto.ca/mirDIP/index.jsp#r) and starBase (http://starbase.sysu.edu.cn/) databases were utilized to predict the upstream miRNAs of target mRNA, and the Venn diagram was used to map the intersection of the upstream downregulated DEmiRNAs for MT1X. The UALCAN portal (http://ualcan.path.uab.edu/analysis-prot.html) was an interactive web resource for us to perform mRNA and miRNA expression of the TCGA (The Cancer Genome Atlas) data set. ONCOMINE (https://www.oncomine.org), translational bioinformatics service, was utilized to evaluate the expression of MTs in AML.

### Cell lines and samples

Human AML cell line THP1 and hematopoietic precursor cell line K562 were purchased from ATCC (Manassas, USA) and maintained in RPMI-1640 (HyClone, USA) supplemented with 10% fetal bovine serum (Gibco, USA) at 37°C with 5% CO2. THP1 cells were treated with doxorubicin (DOX) (Selleck, USA) at different concentrations (0, 0.05, 0.1, 0.5, 1, 5, 10, 20 μg/ml) for 48h. All participants signed informed consent forms, and a total of 20 bone marrow samples (14 AML patient samples and 6 samples from healthy individuals) were obtained between October 2019 and July 2021 from the Xinyang Hospital Affiliated to Zhengzhou University.

### Cell transfection

siNC, siMT1X (siMT1X-1, siMT1X-2, and siMT1X-3), mimic NC, miR-376a-3p mimic, oe-NC and oe-MT1X were purchased from RiboBio (Guangzhou, China). The siMT1X-1 sequence was 5’-GGCTCCTGCAAATGCAAAGAGTGCA, siMT1X-2 sequence was 5’- GAGTGCAAATGCACCTCCTGCAAGA, siMT1X-3 sequence was 5’- CATCTGCAAAGGGACGTCAGACAAG, and the siNC sequence was 5’- AATGGAGTCACCTCGCACTGGACAA. THP1 and K562 cells were plated into 6-well plates at a concentration of 3 × 10^6^ /well for 24 h before transfection with 2 μg oe-MT1X or 40pmol siMT1X or 40pmol miR-376a mimic by electroporation (Bio-rad, USA) following the manufacturer’s instructions.

### RNA extraction and qRT-PCR

Total RNA was extracted from cells using TRIpure Total RNA Extraction Reagent (ELK Biotechnology, Wuhan, China). cDNA was synthesized using an EntiLink™ First Strand cDNA Synthesis Kit (ELK Biotechnology, Wuhan, China). qRT-PCR was conducted using the EnTurbo™ SYBR Green PCR SuperMix. U6 and GAPDH served as internal references for normalization. Primer sequences are listed in Table 1.

**Table 1.** The primer sequences for qRT-PCR in our study

Gene	Forward	Reverse
GAPDH	5ʹCATCATCCCTGCCTCTACTGG3’	5ʹGTGGGTGTCGCTGTTGAAGTC3’
MT1F	5ʹCCACTGCTTCTTCGCTTCTCT3’	5ʹAAGGTTGTCCTGGCATCAGTC3’
MT1G	5ʹCTTCTCGCTTGGGAACTCTAGTC3’	5ʹGGTCAAGATTGTAGCAAAAAACAA3’
MT1H	5ʹCTCGCTTGGGAACTCCAGTC3’	5ʹGTTTTCATCTGACAGCAGGGC3’
MT1X	5ʹCGTGTTTTCCTCTTGATCGG3’	5ʹGCTGCACTTGTCTGACGTCC3’
P65	5ʹCGCATCCAGACCAACAACA3’	5ʹTGCCAGAGTTTCGGTTCAC3’
CyclinD1	5ʹTCCTACTTCAAATGTGTGCAGAAG3’	5ʹCATCTTAGAGGCCACGAACATG3’
miR-376a-3p U6	5ʹGGCATAGAGGAAAATCCACG3’ 5ʹCTCGCTTCGGCAGCACAT3’	5ʹCTCAACTGGTGTCGTGGAGTC3’ 5ʹAACGCTTCACGAATTTGCGT3’

### Western Blot (WB)

The total proteins from cells were solubilized in RIPA lysis buffer (ASPEN, China) and were loaded into the sodium dodecyl sulfate polyacrylamide gel electrophoresis (SDS-PAGE). The proteins were transferred to the polyvinylidene fluoride membranes (Millipore, Germany) and blocked with 5% skim milk powder for 1 h. The membranes were then incubated overnight at 4°C with primary antibody as follows: P-IκBα rabbit monoclonal antibody (ab133462, 1:1000, Abcam, Cambridge, UK), cleaved caspase3 rabbit polyclonal antibody (ab49822, 1:1000, Abcam, Cambridge, UK), Ki-67 rabbit monoclonal antibody (ab92742, 1:500, Abcam, Cambridge, UK), GAPDH rabbit polyclonal antibody (ab37168, 1:10000, Abcam, Cambridge, UK), p65 rabbit monoclonal antibody (#8242, 1:2000, CST, USA), cyclinD1 rabbit monoclonal antibody (#2878, 1:1000, CST, USA), IκB-α rabbit polyclonal antibody (18220-1-AP, 1:2000, Proteintech Group, Inc., USA), and MT1X rabbit polyclonal antibody (OACA02206, 1:500, avivasysbio, USA). Then, the membranes were incubated with horseradish peroxidase-labeled secondary antibody goat anti-rabbit IgG (AS1107, 1:10000, ASPEN, China) at room temperature for 1 h and were washed with PBS containing 0.1% Tween-20 buffer for 10 min 3 times. The protein bands were visualized with an ECL kit (ASPEN, China), and the band intensity was analyzed using AlphaEaseFC software.

### Cell proliferation assay

In vitro cell proliferation was assayed using Cell Counting Kit 8 (CCK8; Beyotime, C0038, China). Briefly, 1 × 10^4^ cells were seeded onto each well of a 96-well plate. Ten μl CCK-8 solution was added to each well at times 0 and 24, 48, 72 and 96 h followed by 4 h more. Absorbance at 450 nm was measured using a Microplate Reader (Diatek, China).

### Cell apoptosis and cell cycle assays

THP1 cells were seeded onto the 24-well plate and evaluated with an Annexin V-FITC/PI Apoptosis Detection kit (BD Biosciences, Germany) via a flow cytometer (BD FACSCalibur, USA). The cells for the cell cycle assay were collected, washed and fixed with 90% cold ethanol for 20 min at 4°C. The cells were incubated with propidium iodide (PI) for 20 min at room temperature. Then, the cells were analyzed using a flow cytometer.

### Dual-luciferase reporter gene assay

The wild-type MT1X (MT1X-WT) and mutant-type MT1X (MT1X-MUT) at binding sites of miR-376a-3p were constructed according to pGL6-miR. THP1 cells were seeded into 24-well plates with a density of 3x10^6^/well and co-transfected with MT1X-WT or MT1X-MUT vector, and miR-376a-3p mimic or mimic NC through electroporation (Bio-rad, USA) following the manufacturer’s instructions. After transfection for 48 h, the dual-luciferase reporter gene assay system (Beyotime, RG028, China) was utilized to measure the luciferase activity.

### Immune histochemistry

Bone marrow samples were fixed in 4% paraformaldehyde solution, paraffin-embedded and sliced into 5-µm-thick sections. Bone marrow samples were stained with specific antibodies against MT1X (17172-1-AP, 1:300 dilution, rabbit polyclonal; Proteintech Group, Inc., USA) and p65 (ab16502, 1:300 dilution, rabbit polyclonal; Abcam, Cambridge, UK) following antigen retrieval. A pre-diluted secondary antibody (Servicebio) was added to the slides for 1 h followed by impact DAB (Beijing Zhongshan Golden Bridge Biotechnology Co., Ltd., China) staining. Slides were counter-stained with hematoxylin and mounted with neutral gum. Samples were evaluated microscopically, and photographs were taken using a 10× eyepiece. Tissue microarrays were imaged at 200× magnifications.

### Statistical analysis

Graphpad Prism 7.0 (Graphpad Software Inc.) was utilized for statistical analysis. The results were presented as mean ± SEM. A two-tailed Student’s t-test was employed to compare differences between only two groups. One-way ANOVA or two-way ANOVA was employed to determine significance when there were three or more groups. Spearman’s correlation analysis was utilized to determine the correlation between protein levels of MT1X and P65 in AML. Values of P < .05 were considered statistically significant differences.

## Data Availability

The data sets used and/or analyzed during the current study are available from the corresponding authors on reasonable request.
